# Clinical and molecular characterization of thrombocytosis in transient abnormal myelopoiesis

**DOI:** 10.1038/s41375-026-02960-z

**Published:** 2026-05-05

**Authors:** Satoshi Saida, Tsutomu Toki, Ritsuko Shimizu, Asahito Hama, Takao Deguchi, Genki Yamato, Rika Kanezaki, Shotaro Iwamoto, Mari Morimoto, Daisuke Hasegawa, Takahiro Ueda, Tomoko Yokosuka, Kyogo Suzuki, Akira Shimada, Yasuhide Hayashi, Etsuro Ito, Kenichiro Watanabe, Kiminori Terui, Hideki Muramatsu

**Affiliations:** 1 Department of Pediatrics, Graduate School of Medicine, Kyoto University, Kyoto, Japan; 2 Department of Pediatrics, Hirosaki University Graduate School of Medicine, Hirosaki, Japan; 3 Department of Molecular Hematology, Tohoku University Graduate School of Medicine, Sendai, Japan; 4 Department of Hematology and Oncology, Children’s Medical Center, Japanese Red Cross Aichi Medical Center Nagoya First Hospital, Nagoya, Japan; 5 Children’s Cancer Center, National Center for Child Health and Development, Tokyo, Japan; 6 Department of pediatrics, Gunma University Graduate School of Medicine, Maebashi, Japan; 7 Department of Pediatrics, Mie University Graduate School of Medicine, Tsu, Japan; 8 Department of Pediatrics, St. Luke’s International Hospital, Tokyo, Japan; 9 Department of Pediatrics, Nippon Medical School, Tokyo, Japan; 10 Department of Hematology/Oncology, Kanagawa Children’s Medical Center, Yokohama, Japan; 11 Department of Pediatrics and Developmental Biology, Institute of Science Tokyo, Tokyo, Japan; 12 Department of Pediatrics, Jichi Medical University Hospital, Shimotsuke, Tochigi, Japan; 13 Department of Hematology/Oncology, Gunma Children’s Medical Center, Shibukawa, Japan; 14 Department of Community Medicine, Hirosaki University Graduate School of Medicine, Hirosaki, Japan; 15 Department of Hematology and Oncology, Shizuoka Children’s Hospital, Shizuoka, Japan; 16 Department of Pediatrics, Nagoya University Graduate School of Medicine, Nagoya, Japan

**Keywords:** Acute myeloid leukaemia, Acute myeloid leukaemia

## To the Editor:

Transient abnormal myelopoiesis (TAM) is a distinctive hematological disorder in neonates with Down syndrome, driven by somatic *GATA1* mutations that promote blast proliferation [1, 2]. Although TAM typically resolves spontaneously, a subset of patients may progress to myeloid leukemia associated with Down syndrome (ML-DS) [3–6]. Clinically, TAM ranges from asymptomatic cases to severe disease with multiorgan dysfunction.

While thrombocytopenia is well recognized in TAM, sporadic reports of thrombocytosis [7, 8] indicate that platelet abnormalities may be more heterogeneous than previously appreciated. In some cohorts, platelet counts have approached 1000 × 10⁹/L [3, 4]. Whether elevated counts increase thrombosis risk, define a distinct clinical subtype, or influence disease progression has not been systematically explored.

To address this gap, we conducted a secondary analysis of the TAM-10 prospective cohort, stratified by platelet count at diagnosis. We hypothesized that thrombocytosis represents a clinically and biologically distinct TAM subgroup, reflecting potential differences in underlying pathophysiology. Detailed methods are provided in the Supplementary Methods.

## Results

### Characteristics of patients with TAM based on platelet count

**Fig. 1:**
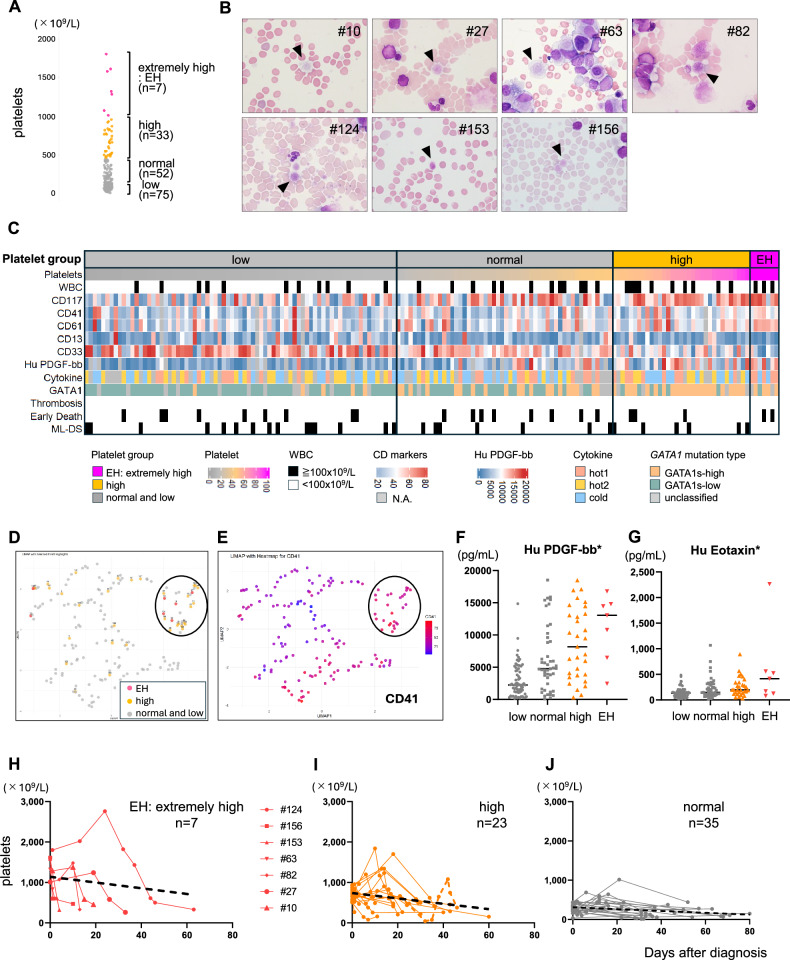
Clinical and molecular features of patients with TAM with thrombocytosis. **A** Distribution of platelet counts at diagnosis in the TAM cohort. **B** Peripheral blood smears from seven representative cases with EH platelet counts, demonstrating prominent giant platelets. **C** Heatmap of clinical and molecular parameters stratified by platelet count group. Rows are ordered by platelet count. Annotations include surface marker expression, cytokine profiles, *GATA1* mutation type, and clinical outcomes. The heatmap was generated using the ComplexHeatmap and circlize packages in RStudio. **D** Surface marker expression profiles in blast cells. UMAP plot of 164 patients with TAM based on expression percentages of six surface antigens (CD7, CD117, CD13, CD33, CD41, and CD61). Pink, orange, and gray dots indicate patients in the EH, high, and normal-to-low platelet count groups, respectively. UMAP was generated using the umap package in RStudio. The circle highlights a loosely defined cluster of patients with EH platelet counts, suggesting similarity in their immunophenotypic profiles. **E** CD41 expression overlaid on UMAP coordinates; red denotes high and blue low expression. **F**, **G** Bar plots of representative cytokines that were significantly elevated in patients with high or EH platelet counts. Serum cytokine levels are compared across four platelet count groups (EH, high, normal, and low) at diagnosis. Of the 27 cytokines analyzed, 15 showed statistically significant differences (p < 0.05); the full list is provided in the main text, Supplementary Fig. 4, and Supplementary Table 4. Statistical analyses were performed using the Kruskal–Wallis test or one-way ANOVA, as appropriate. Asterisks indicate statistically significant differences. Sequential changes in platelet counts in patients with TAM. Temporal changes in platelet counts in patients with EH (**H**), high (**I**), and normal (**J**) platelet levels at diagnosis. The black dashed line represents the linear regression line.

At diagnosis, platelet counts were available for all 167 patients in the TAM-10 cohort (Supplementary Table 1) [9]. Patients were stratified into four groups: ≥1000 × 10⁹/L (extremely high [EH]), 450–999 × 10⁹/L (high), 150–449 × 10⁹/L (normal), and <150 × 10⁹/L (low). Seven (4%) patients were classified as EH, 33 (20%) as high, 52 (31%) as normal, and 75 (45%) as low (Fig. 1A).

Baseline characteristics, including sex, gestational age, and birth weight, did not differ among groups (Supplementary Table 2). In contrast, white blood cell (WBC) counts and peripheral blast percentages increased progressively with higher platelet counts (*p* = 0.0006 and *p* = 0.042, respectively). Alanine aminotransferase (ALT) levels and the frequency of hepatomegaly were also significantly higher in the elevated platelet groups (*p* < 0.0001 and *p* = 0.0027, respectively).

Therapeutic interventions, including low-dose cytarabine, exchange transfusion, and systemic corticosteroids, were similarly distributed across groups. No thrombotic events occured, including among patients in the EH platelet group. Only one EH patient received low-dose aspirin. Rates of early death, late death, and ML-DS development did not differ significantly across groups.

### Morphological and molecular features in patients with TAM with thrombocytosis

Peripheral blood smears from all seven EH patients showed marked thrombocytosis with prominent giant platelets, consistent with increased megakaryocytic activation (Fig. 1B). These features were less evident or absent in the other groups.

An integrated heatmap incorporating clinical parameters, immunophenotype, cytokine levels, and *GATA1* mutation patterns (Supplementary Fig. 1 and Supplementary Table 3) revealed clustering of EH and high platelet cases (Fig. 1C). Patients in the EH and high platelet groups more frequently exhibited elevated WBC counts ( ≥ 100 × 10⁹/L). Notably, short-form GATA1 (GATA1s)-high mutations were more prevalent in patients with higher platelet counts (chi-square and Fisher’s exact tests, both *p* < 0.0001).

### Surface marker expression and cytokine profiles by platelet group

Flow cytometric analysis revealed progressive increases in megakaryocytic and progenitor markers (CD41, CD61, CD117) with rising platelet counts, accompanied by relatively reduced expression of myeloid markers such as CD33 (Supplementary Fig. 2). In the UMAP analysis, patients in the EH group tended to form a loosely defined cluster, suggesting partial similarity in their immunophenotypic profiles (Fig. 1D). The UMAP-based distribution of surface marker expression further supported increased expression of megakaryocytic markers in the EH and high groups (Fig. 1E and Supplementary Fig. 3).

Serum cytokine profiling detected 15 of 27 cytokines (shown in Supplementary Table 4) [10], demonstrating significant differences across platelet count groups (Fig. 1F, G and Supplementary Fig. 4). Patients with EH and high platelet counts exhibited elevated levels of pro-inflammatory and hematopoietic cytokines, including IL-4, IL-7, IL-9, IL-10, PDGF-bb, basic FGF, Eotaxin, G-CSF, IFN-γ, IP-10, MCP-1, RANTES, and TNF-α. Despite this broad cytokine upregulation, the previously established hot1/hot2/cold cytokine categories were not significantly different by platelet group (Fig. 1C and Supplementary Table 2) [10].

### Time-course of platelet counts in patients with TAM

Subsequently, we examined longitudinal changes in platelet counts following diagnosis. In most EH and high-platelet cases, platelet counts peaked approximately 1 month after diagnosis and then declined spontaneously without specific intervention (Fig. 1H–J). Longitudinal platelet trends were generally similar between cytarabine-treated and untreated patients, although early low-dose cytarabine treatment was associated with a modest attenuation of platelet decline (Supplementary Fig. 5). Additionally, several patients later developed transient thrombocytopenia about 1 month after diagnosis, followed by spontaneous recovery within approximately 3 months. However, persistent thrombocytopenia was observed only in cases with leukemic transformation or fatal outcomes (Supplementary Fig. 6).

### Association between *GATA1* mutation pattern and hematological and biological parameters

**Fig. 2:**
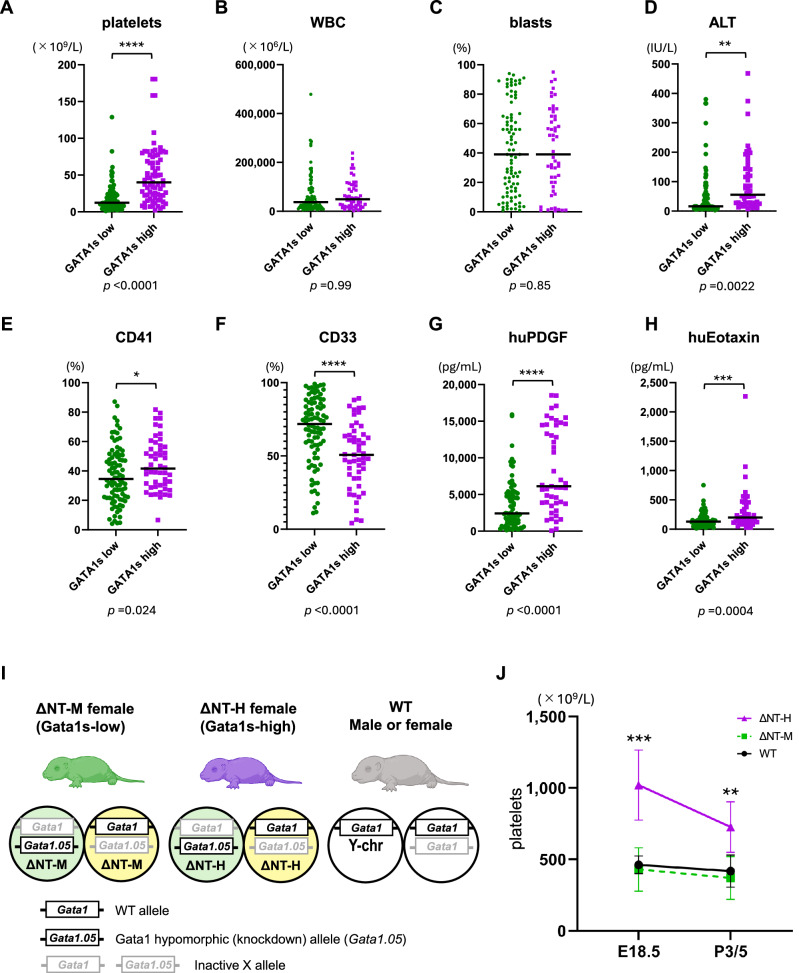
Association between *GATA1* mutation patterns and clinical and biological parameters in patients with TAM. Peripheral blood counts (**A**–**C**), serum alanine aminotransferase (ALT) levels (**D**), surface marker expression on blast cells by flow cytometry (**E**, **F**), and serum cytokine concentrations (**G**, **H**) at diagnosis, stratified by *GATA1* mutation type (GATA1s-low [green] vs GATA1s-high [purple]). Each dot represents an individual patient, and horizontal bars indicate median values. Statistical analyses were performed with the Mann–Whitney U test. Asterisks denote statistical significance (*p < 0.05, **p < 0.01, ***p < 0.001, ****p < 0.0001). **I** Schematic of two types of megakaryocyte progenitors in heterozygous *Gata1.05*/X females carrying a transgene. The *Gata1.05* allele is a targeted knockdown resulting in only ~5% of wild-type Gata1 expression. The green mouse represents a ΔNT-M female (Gata1s-low), the purple mouse represents a ΔNT-H female (Gata1s-high), and the gray mouse represents a wild-type control. Megakaryocyte progenitors with an activated mutant X chromosome are shown in light green, whereas those with an activated wild-type allele are shown in yellow. The inactivated *Gata1* allele resulting from X-chromosome inactivation is indicated in light gray. **J** Comparison of platelet counts among Gata1s-low (ΔNT-M: green), Gata1s-high (ΔNT-H: purple), and wild-type (WT: gray) mice at various time points. Data are presented as mean ± standard deviation. Asterisks denote statistical significance (*p < 0.05, **p < 0.01, ***p < 0.001, ****p < 0.0001).

Based on our previously established classification, patients were stratified into GATA1s-low and GATA1s-high type mutation groups, reflecting the expected level of short-form GATA1 (GATA1s) expression (Supplementary Fig. 1) [11]. As shown in Fig. 2A, platelet counts were significantly higher in patients in the GATA1s-high mutation group compared to those in the GATA1s-low mutation group (*p* < 0.0001). Conversely, there were no significant differences in WBC counts (*p* = 0.99) or blast percentages (*p* = 0.85) between the two groups (Fig. 2B, C). Serum ALT levels were also significantly higher in the GATA1s-high group (Fig. 2D, p = 0.0022). Immunophenotypically, the GATA1s-high group exhibited increased CD41 expression (*p* = 0.024) and reduced CD33 expression (*p* < 0.0001), consistent with megakaryocytic skewing (Fig. 2E, F). Furthermore, GATA1s-high patients exhibited markedly higher serum levels of the hematopoietic cytokine PDGF (Fig. 2G, p < 0.0001), the inflammatory cytokine Eotaxin (Fig. 2H, p = 0.0004), as well as IL-4 (*p* = 0.0008) and IL-7 (*p* = 0.041), supporting a distinct cytokine milieu associated with *GATA1* mutation type.

### Higher *Gata1s* expression correlates with elevated platelet counts in transgenic mice

To validate the role of GATA1s dosage, we analyzed transgenic mouse models expressing high (ΔNT-H) or low (ΔNT-M) levels of Gata1s (Fig. 2I) [12]. In the context of reduced endogenous Gata1 expression, mice with high Gata1s (ΔNT-H) exhibited significantly elevated platelet counts at embryonic day 18.5 and postnatal days 3–5 compared to wild-type and ΔNT-M mice (Fig. 2J). These findings provide experimental support for a causal relationship between GATA1s dosage and thrombopoiesis.

## Discussion

This study demonstrates that thrombocytosis in TAM is associated with characteristic clinical and biological features, including higher blast percentages, liver involvement, megakaryocytic skewing, enrichment of GATA1s-high mutation patterns, and broad cytokine activation. Approximately one-quarter of patients with TAM presented with elevated platelet counts, including a small subset with extreme thrombocytosis. Importantly, thrombocytosis was not associated with thrombotic complications or adverse clinical outcomes, and platelet counts generally declined spontaneously over time.

No comparable cases of extreme thrombocytosis have been reported in large ML-DS cohorts. For example, in AML-D05 (median platelet count: 36 × 10⁹/L [range 3–312]) [13]. AML-D11 (median 49 × 10⁹/L [range 5–250]) [14], and COG AAML1531 (median 40.5 × 10⁹/L [range 3–339]) [15], thrombocytosis was not observed despite the shared clonal origin with TAM. In contrast, thrombocytosis has been described in TAM cohorts, including COG POG-9481 (median 96 × 10⁹/L [range 21–1,100]) [3], COG A2971 (median 125 × 10⁹/L [range 1–958]) [4], and our JPLSG TAM-10 study (median 192 × 10⁹/L [range 1–1,806]). These findings suggest that transient megakaryocytic hyperplasia with thrombocytosis can occur during TAM but does not persist into the leukemic phase of ML-DS, underscoring stage-specific regulatory mechanisms in megakaryopoiesis. Notably, this study is the first to comprehensively characterize the frequency and natural history of thrombocytosis in TAM.

Our analysis of *GATA1* mutation types showed that thrombocytosis was enriched in patients harboring mutations classified as “GATA1s-high” expression types, typically retaining N-terminal transactivation activity. This finding suggests a genotype–phenotype correlation in which specific *GATA1* alterations promote enhanced megakaryocytic proliferation.

Although thrombocytosis in TAM does not appear to influence prognosis with respect to early death or leukemic transformation, it may represent a distinct biological subset that warrants further study. At the same time, the spontaneous resolution of thrombocytosis in most cases underscores the need to avoid unnecessary treatment while maintaining close monitoring.

Several limitations should be noted when interpreting our findings. Whereas we observed concordant associations between *GATA1* mutation patterns and thrombocytosis in a clinical cohort and in mouse models, this study was descriptive and does not establish the underlying molecular mechanisms linking *GATA1* variant type to increased platelet production. Additionally, platelet function was not evaluated directly, and the cellular origin of increased platelet counts, whether derived predominantly from *GATA1*-mutant clones or from residual normal megakaryopoiesis, could not be determined. Although associations with *GATA1* mutation patterns and cytokine profiles were observed, the potential contribution of other genetic, epigenetic, or microenvironmental factors was not fully explored. Finally, whereas this study leveraged a well-characterized prospective cohort, the number of patients with extreme thrombocytosis was limited. Additional studies will help refine the biological interpretation of thrombocytosis in TAM.

In conclusion, our findings demonstrate that thrombocytosis in TAM is associated with distinctive clinical, morphological, and molecular characteristics, including megakaryocytic skewing and enrichment of specific *GATA1* mutation types. These observations highlight the biological heterogeneity within TAM and suggest that platelet count may reflect underlying differences in disease biology, without implying adverse clinical outcomes.

## Supplementary information

Supplemental Data

## Data Availability

No publicly available datasets were generated or analyzed during the current study. Data supporting the findings of this study are available from the corresponding author upon reasonable request.

## References

[1] Hitzler JK, Cheung J, Li Y, Scherer SW, Zipursky A. GATA1 mutations in transient leukemia and acute megakaryoblastic leukemia of Down syndrome. Blood. 2003;101:4301–4. 12586620 10.1182/blood-2003-01-0013

[2] Xu G, Nagano M, Kanezaki R, Toki T, Hayashi Y, Taketani T, et al. Frequent mutations in the GATA-1 gene in the transient myeloproliferative disorder of Down syndrome. Blood. 2003;102:2960–68. 12816863 10.1182/blood-2003-02-0390

[3] Massey GV, Zipursky A, Chang MN, Doyle JJ, Nasim S, Taub JW, et al. A prospective study of the natural history of transient leukemia (TL) in neonates with Down syndrome (DS): Children’s Oncology Group (COG) study POG-9481. Blood. 2006;107:4606–13. 16469874 10.1182/blood-2005-06-2448

[4] Gamis AS, Alonzo TA, Gerbing RB, Hilden JM, Sorrell AD, Sharma M, et al. Natural history of transient myeloproliferative disorder clinically diagnosed in Down syndrome neonates: a report from the Children’s Oncology Group Study A2971. Blood. 2011;118:6752–59. 21849481 10.1182/blood-2011-04-350017 PMC3245202

[5] Yoshida K, Toki T, Okuno Y, Kanezaki R, Shiraishi Y, Sato-Otsubo A, et al. The landscape of somatic mutations in Down syndrome-related myeloid disorders. Nat Genet. 2013;45:1293–9. 24056718 10.1038/ng.2759

[6] Sato T, Yoshida K, Toki T, Kanezaki R, Terui K, Saiki R, et al. Landscape of driver mutations and their clinical effects on Down syndrome–related myeloid neoplasms. Blood. 2024;143:2627–43. 38513239 10.1182/blood.2023022247

[7] Fujihara I, Yanagisawa R, Fukushima Y, Komori K, Ogiso Y, Sakashita K. Thrombocytosis in a newborn with Down syndrome and transient abnormal myelopoiesis. Br J Haematol. 2016;172:314. 26457464 10.1111/bjh.13808

[8] Lee JW, Kim S, Jang PS, Chung NG, Cho B, Kim M. Marked thrombocytosis resulting in pseudohyperkalemia in a neonate with transient abnormal myelopoiesis. Pediatr Blood Cancer. 2021;68:e28986. 33682342 10.1002/pbc.28986

[9] Yamato G, Deguchi T, Terui K, Toki T, Watanabe T, Imaizumi T, et al. Predictive factors for the development of leukemia in patients with transient abnormal myelopoiesis and Down syndrome. Leukemia. 2021;35:1480–4. 33654203 10.1038/s41375-021-01171-y PMC8102190

[10] Yamato G, Tsumura Y, Muramatsu H, Shimada A, Imaizumi T, Tsukagoshi H, et al. Cytokine profiling in 128 patients with transient abnormal myelopoiesis: a report from the JPLSG TAM-10 trial. Blood Adv. 2024;8:3120–9. 38691583 10.1182/bloodadvances.2023011628 PMC11222942

[11] Kanezaki R, Toki T, Terui K, Xu G, Wang R, Shimada A, et al. Down syndrome and GATA1 mutations in transient abnormal myeloproliferative disorder: mutation classes correlate with progression to myeloid leukemia. Blood. 2010;116:4631–8. 20729467 10.1182/blood-2010-05-282426

[12] Shimizu R, Takahashi S, Ohneda K, Engel JD, Yamamoto M. In vivo requirements for GATA-1 functional domains during primitive and definitive erythropoiesis. Embo J. 2001;20:5250–60. 11566888 10.1093/emboj/20.18.5250 PMC125635

[13] Taga T, Watanabe T, Tomizawa D, Kudo K, Terui K, Moritake H, et al. Preserved high probability of overall survival with significant reduction of chemotherapy for myeloid Leukemia in Down syndrome: a nationwide prospective study in Japan. Pediatr Blood Cancer. 2016;63:248–54. 26481183 10.1002/pbc.25789

[14] Taga T, Tanaka S, Hasegawa D, Terui K, Toki T, Iwamoto S, et al. Post-induction MRD by FCM and GATA1-PCR are significant prognostic factors for myeloid leukemia of Down syndrome. Leukemia. 2021;35:2508–16. 33589754 10.1038/s41375-021-01157-w

[15] Hitzler J, Alonzo T, Gerbing R, Beckman A, Hirsch B, Raimondi S, et al. High-dose AraC is essential for the treatment of ML-DS independent of postinduction MRD: results of the COG AAML1531 trial. Blood. 2021;138:2337–46. 34320162 10.1182/blood.2021012206 PMC8662073

