# Supplementary material for: Clinical and molecular characterization of thrombocytosis in transient abnormal myelopoiesis
Source: Leukemia. 2026 May 5;40(7):1574–8. doi: 10.1038/s41375-026-02960-z (PMC13323064; doi:10.1038/s41375-026-02960-z)
Supplement: Supplementary file 1 — Supplemental Data [file 41375_2026_2960_MOESM1_ESM.docx]

**Supplemental Data**

**Materials and methods**

**Patients**

Between May 2011 and February 2014, 167 neonates (89 boys and 78 girls) with TAM were prospectively enrolled in TAM-10, a multicenter observational cohort study conducted by the Japan Pediatric Leukemia/Lymphoma Study Group (JPLSG) under the aegis of the Japan Children’s Cancer Group (JCCG) (UMIN000005418).^1^ Eligibility criteria were: (i) infants with Down syndrome aged <3 months old, presenting with circulating blast cells in the peripheral blood, and (ii) infants without Down syndrome aged <3 months old, with circulating blast cells, harboring somatic trisomy 21 and a *GATA1* mutation. Morphological diagnosis, flow cytometric immunophenotyping, and *GATA1* mutation analysis were performed centrally in accordance with the TAM-10 protocol. Constitutional trisomy 21 was confirmed locally at each participating institution.

This study is a secondary analysis of the TAM-10 prospective observational cohort, in which the patients were stratified by platelet count at diagnosis to evaluate clinical, inflammatory, and genetic features across the spectrum of platelet abnormalities. Written informed consent was obtained from parents or legal guardians before data and biological sample collection. The TAM-10 study was approved by the institutional review boards of all participating centers. This secondary analysis was conducted in accordance with the Declaration of Helsinki and received ethical approval from the Kyoto University Graduate School and Faculty of Medicine and the Kyoto University Hospital Ethics Committee.

***GATA1* genomic sequencing analysis**

*GATA1* mutations were analyzed by Sanger sequencing of genomic DNA and complementary DNA (cDNA) prepared from peripheral blood at diagnosis, as previously described.^2^ Targeted next-generation sequencing was performed in cases where *GATA1* mutations were not detected by Sanger sequencing.

*GATA1* mutations were classified into two groups, “GATA1s-low” and “GATA1s-high,” based on the predicted expression level of the truncated short-form GATA1 (GATA1s) protein, using a modified version of our previously reported classification system^3^, which incorporates the location and mutation type (Supplemental Figure 1). Classification results for each variant are summarized in Supplemental Table 3.

**Cytokine analysis**

Serum concentrations of 27 cytokines were measured using the Bio-Plex cytokine assay (Bio-Rad, Hercules, CA), as previously described.^4^ The cytokines assessed are listed in Supplemental Table 4. Based on the expression profiles, cytokine patterns were categorized into three groups: hot1, hot2, and cold, following previously established criteria.^4^

**Flow cytometric analysis**

Antibodies used for TAM diagnosis using flow cytometry are listed in Supplemental Table 5. Peripheral blood samples were incubated with antibodies for 15 min at room temperature in the dark, followed by red blood cell lysis with FACS Lysing Solution (BD Bioscience, Franklin Lakes, NJ) and a single wash with Dulbecco’s phosphate-buffered saline (Sigma-Aldrich, St Louis, MO). Data acquisition was performed on a FACS Canto II flow cytometer (BD Biosciences), and analyses were conducted using FACS DIVA software (BD Biosciences).

**Mice**

Two independent transgenic lines were used: ΔNT-H and ΔNT-M, both expressing *Gata1s* under the control of the *Gata1* hematopoietic regulatory domain (G1HRD).^5^ In fetal liver cells, ΔNT-H mice express *Gata1s* mRNA at levels far exceeding endogenous *Gata1*, whereas ΔNT-M mice express it at levels comparable to endogenous expression.^5^

To generate experimental mice, ΔNT-H or ΔNT-M transgenic males were crossed with heterozygous Gata1.05/X females. The Gata1.05 allele is a targeted knockdown resulting in only ~5% of wild-type Gata1 expression.^6^ Female offspring carrying a transgene (ΔNT-H or ΔNT-M) were used for analysis. Because the *GATA1* gene is located on the X chromosome, female offspring exhibit two types of megakaryocyte progenitors due to X-chromosome inactivation: one type carries an activated mutant X chromosome, whereas the other carries an inactivated mutant allele. In the former, the transgene-derived Gata1s protein is expressed in the context of reduced endogenous Gata1 expression. In contrast, the latter expresses endogenous wild-type Gata1 and transgene-derived Gata1s proteins (Figure 2I).

The *Gata1*.05 allele was identified by genomic polymerase chain reaction (PCR) using a pair of primers specific to the neomycin resistance gene.^6^ *Gata1* transgenes were genotyped with PCR, using primer sets designed to amplify the fusion sites of transgene insertion in each of the two transgenic mouse lines, as previously described.^7^ Blood samples were collected from the trunk following decapitation of embryos/newborns. Platelet counts were determined using Celltac-α MEK6450 autohemocytometers (Nihon Koden, Japan). All animal experimental procedures were approved by the Institutional Animal Experiment Committee of Tohoku University and conducted in accordance with the university’s regulations for animal experiments (2015MdA-001, 2018MdA-058, 2018MdA-245, 2019MdA-289).

**Statistical analysis**

All statistical analyses were performed using GraphPad Prism version 9 (GraphPad Software, San Diego, CA). Graphs were generated using RStudio (Posit Software, Boston, MA). Heatmaps were generated using the ComplexHeatmap and circlize packages in R (RStudio, Posit Software), without clustering. Dimensionality reduction and visualization were performed with Uniform Manifold Approximation and Projection (UMAP) using the umap package in R. Comparisons among the four platelet count groups were performed with one-way ANOVA or the Kruskal–Wallis test, as appropriate. Categorical variables were analyzed with the chi-square or Fisher’s exact test. Cytokine and CD surface marker expression levels were compared using one-way ANOVA or the Kruskal–Wallis test, depending on the data distribution. Two-tailed p-values <0.05 were considered indicative of statistical significance.

**Supplemental Table 1. White blood cell count, percentage of blasts, and platelet count at diagnosis** **in patients with transient abnormal myelopoiesis**

| UPN | WBC (×10^9^/L) | Blast (%) | Platelet (×10^9^/L) |
| --- | --- | --- | --- |
| 1 | 37.5 | 1.3 | 68 |
| 2 | 201.1 | 60.8 | 530 |
| 3 | 104.7 | 51 | 338 |
| 4 | 87.82 | 89 | 421 |
| 5 | 4.7 | 11.7 | 26 |
| 6 | 117.35 | 31 | 655 |
| 7 | 10.3 | 6 | 189 |
| 8 | 93.7 | 65 | 236 |
| 9 | 49.7 | 46 | 268 |
| 10 | 47.2 | 47 | 1287 |
| 11 | 10 | 34.5 | 748 |
| 12 | 2.4 | 1 | 174 |
| 13 | 20.06 | 11 | 145 |
| 14 | 149.7 | 86.5 | 365 |
| 15 | 62.21 | 26.3 | 200 |
| 16 | 290.6 | 94 | 242 |
| 17 | 139.6 | 90 | 121 |
| 18 | 9.2 | 27 | 31 |
| 19 | 176 | 24.8 | 841 |
| 20 | 30 | 5 | 143 |
| 21 | 40.9 | 42 | 204 |
| 22 | 56.7 | 56 | 320 |
| 23 | 20.73 | 28 | 32 |
| 24 | 45.58 | 6 | 84 |
| 25 | 6.8 | 10 | 68 |
| 26 | 11.13 | 19 | 68 |
| 27 | 72.5 | 50 | 1015 |
| 28 | 45.4 | 41 | 522 |
| 29 | 88.3 | 95.5 | 141 |
| 30 | 16.7 | 9.5 | 129 |
| 31 | 8.7 | 3.5 | 32 |
| 32 | 59 | 49 | 227 |
| 33 | 17.6 | 30 | 329 |
| 34 | 63.3 | 55 | 590 |
| 35 | 20.4 | 31 | 223 |
| 36 | 93.1 | 66.8 | 608 |
| 37 | 36.1 | 26 | 143 |
| 38 | 37.59 | 1.5 | 260 |
| 39 | 36.7 | 32 | 960 |
| 40 | 285.6 | 91 | 136 |
| 41 | 53.2 | 20 | 549 |
| 42 | 28.15 | 3.3 | 270 |
| 43 | 14.4 | 23.5 | 400 |
| 44 | 114.77 | 56.5 | 485 |
| 45 | 190 | 56 | 495 |
| 46 | 38.3 | 64 | 138 |
| 47 | 23.8 | 11 | 425 |
| 48 | 25 | 70 | 138 |
| 49 | 148.696 | 70 | 314 |
| 50 | 176 | 68 | 433 |
| 51 | 39.38 | 17 | 318 |
| 52 | 8.2 | 4 | 105 |
| 53 | 52.9 | 72 | 662 |
| 54 | 57.8 | 74.5 | 86 |
| 55 | 16.14 | 29 | 47 |
| 56 | 76.2 | 56 | 146 |
| 57 | 10.1 | 20.5 | 118 |
| 58 | 159 | 24 | 420 |
| 59 | 45.4 | 65 | 771 |
| 60 | 90.3 | 49 | 665 |
| 61 | 21.5 | 87 | 71 |
| 62 | 26.51 | 39 | 50 |
| 63 | 215.6 | 95 | 1325 |
| 64 | 22.4 | 2.5 | 153 |
| 65 | 176.1 | 27 | 58 |
| 66 | 21 | 38 | 46 |
| 67 | 119 | 65 | 668 |
| 68 | 19.1 | 77.5 | 29 |
| 69 | 81.2 | 79.8 | 275 |
| 70 | 17.5 | 31 | 435 |
| 71 | 113.2 | 33 | 146 |
| 72 | 24.1 | 1 | 438 |
| 73 | 24.7 | 2 | 66 |
| 74 | 91.9 | 87.4 | 198 |
| 75 | 26.4 | 33 | 923 |
| 76 | 54.44 | 51 | 80 |
| 77 | 52.1 | 24 | 135 |
| 78 | 175 | 92.5 | 107 |
| 79 | 14.27 | 6 | 59 |
| 80 | 116.5 | 80 | 100 |
| 81 | 109 | 52 | 708 |
| 82 | 107.487 | 84 | 1078 |
| 83 | 28.38 | 20 | 768 |
| 84 | 102.1 | 82 | 189 |
| 85 | 5.13 | 1.2 | 111 |
| 86 | 92 | 78.5 | 874 |
| 87 | 17.87 | 61 | 10 |
| 88 | 59 | 52 | 936 |
| 89 | 7.41 | 0.5 | 777 |
| 90 | 155.9 | 90 | 362 |
| 91 | 69.6 | 45 | 831 |
| 92 | 98.7 | 93 | 84 |
| 93 | 127 | 86 | 45 |
| 94 | 51.42 | 3.5 | 128 |
| 95 | 17.4 | 23.5 | 368 |
| 96 | 478.69 | 89 | 504 |
| 97 | 24.9 | 23 | 268 |
| 98 | 173.4 | 79.5 | 254 |
| 99 | 4.8 | 3.5 | 66 |
| 100 | 16.05 | 4 | 93 |
| 101 | 53.5 | 59 | 41 |
| 102 | 11.71 | 14 | 92 |
| 103 | 8.95 | 14 | 287 |
| 104 | 8.88 | 20 | 479 |
| 105 | 43 | 64 | 72 |
| 106 | 8.4 | 6 | 122 |
| 107 | 54.91 | 81 | 141 |
| 108 | 129.3 | 81.5 | 208 |
| 109 | 59.2 | 23 | 114 |
| 110 | 29.1 | 39 | 62 |
| 111 | 12.8 | 2.5 | 73 |
| 112 | 119.4 | 61 | 231 |
| 113 | 15.6 | 29 | 114 |
| 114 | 178.5 | 37.5 | 420 |
| 115 | 15.78 | 5 | 201 |
| 116 | 26.9 | 1 | 104 |
| 117 | 63.7 | 70 | 688 |
| 118 | 8.6 | 2 | 191 |
| 119 | 5.77 | 3 | 21 |
| 120 | 238.45 | 58 | 503 |
| 121 | 47.86 | 42 | 237 |
| 122 | 24.7 | 6 | 70 |
| 123 | 86.4 | 88.5 | 810 |
| 124 | 61 | 62.7 | 1806 |
| 125 | 10.99 | 47 | 61 |
| 126 | 27.12 | 35 | 480 |
| 127 | 11.64 | 17 | 222 |
| 128 | 14.1 | 19 | 27 |
| 129 | 55.1 | 14 | 824 |
| 130 | 36.82 | 37 | 373 |
| 131 | 12.7 | 1 | 80 |
| 132 | 166 | 82 | 70 |
| 133 | 33 | 54 | 344 |
| 134 | 88.4 | 66 | 329 |
| 135 | 275.99 | 90 | 75 |
| 136 | - | - | - |
| 137 | 144.7 | 89.5 | 71 |
| 138 | 90.5 | 46 | 91 |
| 139 | 26 | 30 | 240 |
| 140 | 19.3 | 10.5 | 552 |
| 141 | 48.7 | 56 | 85 |
| 142 | 30.3 | 19 | 81 |
| 143 | 81.2 | 56 | 57 |
| 144 | 18.23 | 7.3 | 90 |
| 145 | 43.1 | 56.5 | 508 |
| 146 | 5.74 | 13 | 168 |
| 147 | 39.1 | 57 | 460 |
| 148 | 38.15 | 37 | 226 |
| 149 | 268.7 | 88 | 120 |
| 150 | 35.4 | 58 | 147 |
| 151 | 20.65 | 2 | 99 |
| 152 | 16.42 | 53 | 23 |
| 153 | 25.6 | 12 | 1582 |
| 154 | 32.2 | 16 | 77 |
| 155 | 174.9 | 78.5 | 823 |
| 156 | 172 | 60 | 1613 |
| 157 | 21.62 | 29.9 | 238 |
| 158 | 21.9 | 56 | 178 |
| 159 | 6.9 | 2 | 44 |
| 160 | 11.3 | 9 | 42 |
| 161 | 87.3 | 85 | 253 |
| 162 | 12.1 | 1 | 121 |
| 163 | 50.1 | 62 | 192 |
| 164 | 7.65 | 6 | 46 |
| 165 | 16.6 | 32 | 158 |
| 166 | 14 | 10 | 99 |
| 167 | 8.3 | 3 | 440 |
| 168 | 17.1 | 16 | 139 |

Note: Case 136 was later deemed ineligible and is excluded from the analysis. The case number is retained in the table for consistency.

**Supplemental Table 2. Clinical characteristics of TAM patients based on platelet count at diagnosis**

|  | | | Platelets <150 ×10^9/L  n = 75 | | 150–450 ×10^9/L  n = 52 | | 450–1,000 ×10^9/L  n = 33 | ≥1,000 ×10^9/L  n = 7 | *P*-value |
| --- | --- | --- | --- | --- | --- | --- | --- | --- | --- |
| Sex (male:female) | | | 40:35 | | 27:25 | | 16:17 | 5:2 | 0.7423 |
| Median gestational age, weeks (range) | | | 37 (30–40) | | 37 (29–40) | | 37 (32–39) | 36 (29–38) | 0.2230 |
| Median birth weight, grams (range) | | | 2,648 (1,066–3,714) | | 2,516 (1116–3678) | | 2,612 (1,541–3,498) | 2,486 (1,438–3,118) | 0.5413 |
| Median age at diagnosis, days (range) | | | 1 (0–48) | | 1 (0–67) | | 0 (0–25) | 0 (0–4) | 0.0822 |
| **Median WBC count at diagnosis,** ×**10^9^/L (range)** | | | **24.7 (4.8–285.6)** | | **39.3 (2.4–290.6)** | | **63.3 (7.4–478.6)** | **72.5 (25.6–215.6)** | **0.0006** |
| **Median blast percentage in PB at diagnosis, % (range)** | | | **27.0 (1–95.5)** | | **37.0 (1–94)** | | **52.0 (0.5–89.0)** | **60.0 (12.0–95.0)** | **0.0422** |
| **Median platelet count at diagnosis,** ×**10^9^/L (range)** | | | **81 (10-147)** | | **257 (153-440)** | | **665 (460–960)** | **1,287 (825–1,806)** | **<0.0001** |
| Direct bilirubin, mg/dl, median (range) | | | 0.8 (0.0–12.3) | | 0.7 (0.04–3.14) | | 0.7 (0.1–3.0) | 0.9 (0.2–2.2) | 0.6404 |
| **ALT, IU/L, median (range)** | | | **15.0 (3–366)** | | **32.5 (7–468)** | | **79.0 (12–198)** | **62.0 (11–222)** | **<0.0001** |
| **Hepatomegaly, cm, median (range)**** | | | **1.5 (0–7)** | | **3.0 (0–7)** | | **3.5 (0–7)** | **4.0 (3–8)** | **0.0027** |
| Organ hemorrhage, n (%) | | | 5 (6.8) | | 5 (9.8) | | 3 (9.0) | 1 (14.2) | 0.8662 |
| Thrombosis, n (%) | | | 0 (0) | | 0 (0) | | 0 (0) | 0 (0) | - |
| Therapeutic interventions for TAM | | |  | |  | |  |  |  |
| Low dose cytarabine, n (%) | | | 18 (24.6) | | 17 (33.3) | | 15 (45.4) | 2 (28.5) | 0.1706 |
| Exchange blood transfusion, n (%) | | | 8 (10.9) | | 7 (13.7) | | 3 (9.0) | 0 (0) | 0.6348 |
| Systemic steroid therapy, n (%) | | | 11 (14.6) | | 11 (21.2) | | 5 (15.2) | 3 (42.8) | 0.2579 |
| Therapeutic interventions for thrombocytosis | | |  | |  | |  |  |  |
| Low-dose aspirin, n (%) | | | - | | - | | 0 (0) | 1 (14.3) | - |
| Events, n | | |  | |  | |  |  |  |
| Early death (<9 months of age), n (%) | | | 9 (12.3) | | 8 (15.7) | | 3 (9.1) | 2 (28.6) | 0.5233 |
| Later phase death (after 9 months), n (%) | | | 5 (6.8) | | 1 (2.0) | | 3 (9.1) | 0 (0) | 0.4316 |
| Leukemia development, n (%) | | | 12 (16.4) | | 7 (13.7) | | 6 (18.2) | 0 (0) | 0.6469 |
| Cytokine pattern | | |  | |  | |  |  |  |
| Hot1, n / number of measured cases (%) | | | 12/54 (22.2) | | 12/39 (30.8) | | 8/28 (28.6) | 5/7 (71.4) | 0.0602 |
| Hot2, n / number of measured cases (%) | | | 23/54 (42.6) | | 10/39 (25.6) | | 9/28 (32.1) | 0/7 (0.0) | 0.0829 |
| Cold, n / number of measured cases (%) | | | 19/54 (35.2) | | 17/39 (43.6) | | 11/28 (30.3) | 2/7 (28.6) | 0.7820 |
| *GATA1* mutation pattern | | |  | |  | |  |  | **<0.0001** |
| GATA1s–high | | | 5 | | 20 | | 23 | 4 |  |
| GATA1s–low | | | 58 | | 27 | | 6 | 1 |  |
|  |  |  | |  | |  |  |  |  |
|  |  |  | |  | |  |  |  |  |

**Supplemental Table 3. *GATA1* mutation details and classification by predicted expression type in patients with transient abnormal myelopoiesis**

| UPN | *GATA1* mutation | | The number of mutant clones | Mutation Type | Expression type |
| --- | --- | --- | --- | --- | --- |
|  | Nucleotide change | Amino acid change |  |  |  |
| 1 | c.3G>A | - | 1 | LOM | High |
| 2 | c.47_78del32 | p.Pro16Hisfs*13 | 1 | PTC1 | Low |
| 3 | c.220+1G>A | - | 1 | SE | High |
| 4 | c.90_91delAG | p.Val32Phefs*7 | 1 | PTC1 | Low |
| 5 | not detected | - | 0 | - | - |
| 6 | c.220+1G>A | - | 1 | SE | High |
| 7 | c.115G>T | p.Glu39* | 1 | PTC1 | Low |
| 8 | c.90_91delAG | p.Val32Phefs*7 | 1 | PTC1 | Low |
| 9 | c.174_175ins14 | p.Ala59Trpfs*83 | 1 | PTC 2 | Low |
| 10 | c.101dupT | p.Ser36Leu*4 | 1 | PTC1 | Low |
| 11 | c.1_10del10 | - | 1 | LOM | High |
| 12 | c.212_220del12 | - | 1 | SE | High |
| 13 | c.149_150insA | p.Ser51Glufs*17 | 1 | PTC1 | Low |
| 14 | c.49_50delCA | p.Gln17Val*22 | 1 | PTC1 | Low |
| 15 | c.[137_138CC>AA;143C>T] | p.Ser46* | 1 | PTC1 | Low |
| 16 | c.8dupT | p.Gly5Trpfs*35 | 1 | PTC1 | Low |
| 17 | c.173_174ins[T;161_173] | p.Ala59Profs*89 | 1 | PTC 2 | Low |
| 18 | c.162_183delinsAG | p.Ala55Valfs*6 | 1 | PTC1 | Low |
| 19 | c.21_221-168del544 | - | 1 | SE | High |
| 20 | c.89C>G | p.Ser30* | 1 | PTC1 | Low |
| 21 | c.97_101delTTCTT | p.Phe33Profs*5 | 1 | PTC1 | Low |
| 22 | c.83dupC | p.Glu29Argfs*11 | 1 | PTC1 | Low |
| 23 | c.205_217del13 | p.Tyr69Glnfs*64 | 1 | PTC2 | Low |
| 24 | c.149_150delCG | p.Pro50Glnfs*17 | 2 | PTC1 | Multiple mutation |
|  | c.49dupC | p.Gln17Profs*23 |  | PTC1 |  |
| 25 | c.135_153del19 | p.Ser46Glnfs*85 | 1 | PTC2 | Low |
| 26 | c.140_174del35 | p.Ser47Cysfs*9 | 1 | PTC1 | Low |
| 27 | c.165_184dup20 | p.Tyr62Leufs*82 | 2 | PTC2 | Multiple mutation |
|  | c.3G>A | - |  | LOM |  |
| 28 | c.220+2T>C | - | 1 | SE | High |
| 29 | c.231_232dupGT | - | 1 | - | Unclassified |
| 30 | c.153_174dup22 | p.Ala59Hisfs*16 | 1 | PTC1 | Low |
| 31 | c.149_150insC | p.Ser51Glufs*17 | 1 | PTC1 | Low |
| 32 | c.189C>A | pTyr63* | 1 | PTC1 | Low |
| 33 | c.-19-1G>A | - | 1 | SE | High |
| 34 | c.197_220+1del25 | - | 1 | SE | High |
| 35 | c.220+1G>C | - | 1 | SE | High |
| 36 | c.90_91delAG | p.Val32Phefs*7 | 1 | PTC1 | Low |
| 37 | c.89_95dup7 | p.Phe33Argfs*9 | 1 | PTC1 | Low |
| 38 | c.166_220+23del78 | - | 1 | SE | High |
| 39 | c.1A>G | - | 2 | LOM | Multiple mutation |
|  | c.121_146del26 | p.Leu41Profs*18 |  | PTC1 |  |
| 40 | c.153_154ins10 | pThr52Cysfs*19 | 1 | PTC1 | Low |
| 41 | c.-19-1G>A | - | 1 | SE | High |
| 42 | c.220+3delinsCTACAGAC | - | 1 | SE | High |
| 43 | c.-9-285_152del451 | - | 1 | SE | High |
| 44 | c.1A>G | - | 1 | LOM | High |
| 45 | c.220G>T | - | 1 | SE | High |
| 46 | c.18_34del17 | p.Gly7Argfs*27 | 1 | PTC1 | Low |
| 47 | c.-19-29_-19-6del24 | - | 1 | SE | High |
| 48 | c.212_220+5del14 | - | 1 | SE | High |
| 49 | c.220+2_220+3insGT | - | 1 | SE | High |
| 50 | c.219A>G | - | 1 | SE | High |
| 51 | c.186C>G | p.Tyr62* | 1 | PTC1 | Low |
| 52 | not detected | - | 0 | - | - |
| 53 | c.220+1G>A | - | 1 | SE | High |
| 54 | c.182_195dup14 | p.Ala66Profs*76 | 1 | PTC2 | Low |
| 55 | c.150_160dup11 | p.Thr54Argfs*87 | 1 | PTC2 | Low |
| 56 | c.150_163deiinsTGGCAGCTGCCC | p.Ser51Glyfs*16 | 1 | PTC1 | Low |
| 57 | c.44_48dupTCCCC | p.Gln17Serfs*122 | 1 | PTC2 | Low |
| 58 | c.174delinsCTACTGGCCGCT | p.Ala59Tyrfs*82 | 1 | PTC2 | Low |
| 59 | c.220+2T>A | - | 1 | SE | High |
| 60 | c.220+1G>T | - | 1 | SE | High |
| 61 | c.179_182dupTGGC | p.Tyr62Glyfs*7 | 1 | PTC1 | Low |
| 62 | c.173delC | p.Ala58Glyfs*79 | 1 | PTC2 | Low |
| 63 | c.1A>T | - | 1 | LOM | High |
| 64 | c.1A>G | - | 1 | LOM | High |
| 65 | c.185_195del11 | p.Tyr62Cysfs*2 | 1 | PTC1 | Low |
| 66 | c.150delG | p.Ser51Alafs*86 | 1 | PTC2 | Low |
| 67 | c.-19-160_161del340 | - | 1 | SE | High |
| 68 | c.81_82delAC | p.Pro28Argfs*11 | 1 | PTC1 | Low |
| 69 | c.1A>T | - | 1 | LOM | High |
| 70 | c.231_249del19 | - | 2 | - | Multiple mutation |
|  | c.220+3A>C | - |  | SE |  |
| 71 | c.158_173dup16 | p.Ala59Hisfs*14 | 1 | PTC1 | Low |
| 72 | not detected | - | 0 | - | - |
| 73 | c.161_173dup13 | p.Ala59Argfs*13 | 1 | PTC1 | Low |
| 74 | c.46_49dupCCCC | p.Glu17Profs*24 | 1 | PTC1 | Low |
| 75 | c.[-11_15delinsTCGCAGGTTAA;19G>A] | - | 1 | LOM | High |
| 76 | c.183_186delCTAC | p.Tyr62Thrfs*74 | 1 | PTC2 | Low |
| 77 | c.140_149dup10 | p.Ser51Hisfs*20. | 1 | PTC1 | Low |
| 78 | c.90_91delAG | p.Val32Phefs*7 | 2 | PTC1 | Multiple mutation |
|  | c.184_187dupTACT  ) | p.Tyr63Leufs*6 |  | PTC1 |  |
| 79 | c.154_162delinsGGTG | p.Thr52Glyfs*14 | 2 | PTC1 | Multiple mutation |
|  | c.151_168delinsT | p.Ser51Cysfs*11 |  | PTC1 |  |
| 80 | c.150delG | p.Ser51Alafs*86 | 1 | PTC2 | Low |
| 81 | c.-19_4del23 | - | 1 | LOM | High |
| 82 | c.219A>G | - | 1 | SE | High |
| 83 | c.1A>G | - | 1 | LOM | High |
| 84 | c.1A>G | - | 2 | LOM | Multiple mutation |
|  | c.174_188dup15 | p.Tyr63* |  | PTC1 |  |
| 85 | c.-19-1G>A | - | 1 | SE | High |
| 86 | c.220+1G>C | - | 1 | SE | High |
| 87 | c.5_14del10 | p.Glu2Alafs*132 | 1 | PTC2 | Low |
| 88 | c.220G>A | - | 1 | SE | High |
| 89 | c.-19-33_-19-15del19 | - | 1 | SE | High |
| 90 | c.-4_8delinsGAC | - | 1 | LOM | High |
| 91 | c.231_232dupGT | - | 1 | - | Unclassified |
| 92 | c.90_91delAG | p.Val32Phefs*7 | 1 | PTC1 | Low |
| 93 | c.151_161dup11 | p.Ala56Glnfs*85 | 1 | PTC2 | Low |
| 94 | c.174_188dup15 | p.Tyr63* | 1 | PTC1 | Low |
| 95 | c.-19-1G>A | - | 1 | SE | High |
| 96 | c.153-183dup31 | p.Tyr62Hisfs*16 | 1 | PTC1 | Low |
| 97 | c.189C>A | p.Tyr63* | 1 | PTC1 | Low |
| 98 | c.90_91delAG | p.Val32Phefs*7 | 1 | PTC1 | Low |
| 99 | c.90_91delAG | p.Val32Phefs*7 | 2 | PTC1 | Multiple mutation |
|  | c.159_173dup15[insT] | p.Ala59Hisfs*14 |  | PTC1 |  |
| 100 | c.174_193dup20 | p.Asp65Glyfs*79 | 1 | PTC2 | Low |
| 101 | c.154_170dup17 | p.Ala58Glnfs*85 | 1 | PTC2 | Low |
| 102 | c.121_131del11 | p.Leu41Serfs*23 | 1 | PTC1 | Low |
| 103 | c.186C>A | p.Tyr62* | 1 | PTC1 | Low |
| 104 | c.-19-1G>A | - | 2 | SE | Multiple mutation |
|  | c.181_187dup7[insAAGT] | p.Tyr63* |  | PTC1 |  |
| 105 | c.192_193dupGG | p.Asp65Glyfs*73 | 1 | PTC2 | Low |
| 106 | c.229_357del129 | p.77_119del43 | 1 | - | Unclassified |
| 107 | c.216_220+3delCCCAGGTA | - | 1 | SE | High |
| 108 | c.49C>T | p.Gln17* | 1 | PTC1 | Low |
| 109 | c.150_168delinsTGT | p.Ser51Valfs*81 | 1 | PTC2 | Low |
| 110 | c.124_127dupGATG | p.Ala43Glyfs*26 | 1 | PTC1 | Low |
| 111 | c.149_150ins A | p.Ser51Glufs*17 | 2 | PTC1 | Multiple mutation |
|  | c.171dupT | p.Ala58Cysfs*10 |  | PTC1 |  |
| 112 | c.-19-504_93del616 | - | 1 | SE | High |
| 113 | c.146_173dup28[insCCCCCCC] | p.Ala59Profs*90 | 2 | PTC2 | Multiple mutation |
|  | c.220+1G>C | - |  | SE |  |
| 114 | not tested | - | - | - | - |
| 115 | c.144_198dup55 | p.Asp65Valfs89* | 1 | PTC2 | Low |
| 116 | c.105dupC | p.Ser36Leufs*4 | 1 | PTC1 | Low |
| 117 | c.220G>A | - | 1 | SE | High |
| 118 | c.220_220+9del10 | - | 1 | SE | High |
| 119 | c.189delC | p.Tyr63* | 1 | PTC1 | Low |
| 120 | c.-19-3C>G | - | 1 | SE | High |
| 121 | c.108_109delTG | p.Gly37Alafs*2 | 1 | PTC1 | Low |
| 122 | c.174_185dup12 | p.Tyr62* | 1 | PTC1 | Low |
| 123 | c.1A>G | - | 1 | LOM | High |
| 124 | c.220+1G>A | - | 1 | SE | High |
| 125 | c.176_177insTCTGGGC | p.Ala61Glyfs*9 | 1 | PTC1 | Low |
| 126 | c.90_91delAG | p.Val32Phefs*7 | 1 | PTC1 | Low |
| 127 | c.3G>A | - | 1 | LOM | High |
| 128 | c.150_159dup10[insGCAGCTG] | p.Thr54Alafs*89 | 1 | PTC2 | Low |
| 129 | c.220G>A | - | 1 | SE | High |
| 130 | c.220G>A | - | 1 | SE | High |
| 131 | c.189C>A | p.Tyr63* | 1 | PTC1 | Low |
| 132 | c.89C>A | p.Ser30* | 1 | PTC1 | Low |
| 133 | c.152_153insGTGGGAG | p.Ser51Argfs9* | 1 | PTC1 | Low |
| 134 | c.108_177dup70 | p.Leu60Trpfs*3 | 1 | PTC1 | Low |
| 135 | c.207_208delCA | p.Tyr69* | 1 | PTC1 | Low |
| 136 | - | - | - | - | - |
| 137 | c.183_195del13 | p.Tyr62Leufs*71 | 1 | PTC2 | Low |
| 138 | c.221-67_433del280 | - | 1 | - | Unclassified |
| 139 | c.90_91delAG | p.Val32Phefs*7 | 1 | PTC1 | Low |
| 140 | c.54dupT | p.Val19Cysfs*21 | 1 | PTC1 | Low |
| 141 | c.90_91delAG | p.Val32Phefs*7 | 1 | PTC1 | Low |
| 142 | c.187dupT | p.Tyr63Leufs*5 | 1 | PTC1 | Low |
| 143 | c.159_189del31 | p.Thr54Glyfs*73 | 1 | PTC2 | Low |
| 144 | c.90_91delAG | p.Val32Phefs*7 | 1 | PTC1 | Low |
| 145 | c.151_161dup11 | p.Ala56Glnfs*85 | 2 | PTC2 | Multiple mutation |
|  | c.93_94delGG | p.Val32Phefs*7 |  | PTC1 |  |
| 146 | c.170_174dupCTGCT | pAla59Leufs*80 | 1 | PTC2 | Low |
| 147 | c.220+1G>A | - | 1 | SE | High |
| 148 | c.46_49dupCCCC | pGln17Profs*24 | 1 | PTC1 | Low |
| 149 | c.150delG | p.Ser51Alafs*86 | 1 | PTC2 | Low |
| 150 | c.180_187dup8 | p.Tyr63Trpfs*77 | 1 | PTC2 | Low |
| 151 | c.41_72del32 | p.Pro14Leufs*15 | 1 | PTC1 | Low |
| 152 | c.182_189dup8 | p.Arg64Profs*76 | 1 | PTC2 | Low |
| 153 | c.220+3A>C | - | 1 | SE | High |
| 154 | c.181_185dup5[insGT] | p.Tyr62* | 1 | PTC1 | Low |
| 155 | c.183_193del11 | p.Tyr62Argfs*2 | 1 | PTC1 | Low |
| 156 | c.231_241delinsATGGAGGGGA | p.77_119del43 | 1 | - | Unclassified |
| 157 | c.220+1G>A | - | 1 | SE | High |
| 158 | c.38_39delAG | p.Glu13Alafs*26 | 1 | PTC1 | Low |
| 159 | c.90_91delAG | p.Val32Phefs*7 | 1 | PTC1 | Low |
| 160 | c.348_419delinsCTGACCCTGGGACTC | p.Pro117* | 2 | - | Multiple mutation |
|  | c.187dupT | p.Tyr63Leufs*5 |  | PTC1 |  |
| 161 | c.90_91delAG | p.Val32Phefs*7 | 1 | PTC1 | Low |
| 162 | c.-19-1G>A | - | 1 | SE | High |
| 163 | c.15_28del14 | p.Leu6Aspfs*29 | 1 | PTC1 | Low |
| 164 | c.2T>C | - | 1 | LOM | High |
| 165 | c.157_184dup28 | p.Tyr62Cysfs*15 | 1 | PTC1 | Low |
| 166 | c.182_195dup14 | p.Ala66Profs*76 | 1 | PTC2 | Low |
| 167 | c.1A>G | - | 2 | LOM | Multiple mutation |
|  | c.5A>G | p.Glu2Gly |  | - |  |
| 168 | c.81_82delAC | p.Pro28Argfs*11 | 1 | PTC1 | Low |
|  | | | | | |

LOM, loss of the first methionine; PTC, premature termination codon; SE, splicing error

Note: Case 136 was later deemed ineligible and is excluded from the analysis. The case number is retained in the table for consistency.

Modified from Yamato et al. *Leukemia*. 2021.

**Supplemental Table 4. Cytokines measured in serum samples from patients with transient abnormal myelopoiesis**

| Cytokine |
| --- |
| IL-1b |
| IL-1ra |
| IL-2 |
| IL-4 |
| IL-5 |
| IL-6 |
| IL-7 |
| IL-8 |
| IL-9 |
| IL-10 |
| IL-12 |
| IL-13 |
| IL-15 |
| IL-17 |
| Eotaxin |
| PDGF-bb |
| basic FGF |
| G-CSF |
| GM-CSF |
| IFN-r |
| IP-10 |
| MCP-1(MCAF) |
| MIP-1a |
| MIP-1b |
| RANTES |
| TNF-a |
| VEGF |

Cytokine levels were measured using the Bio-Plex multiplex cytokine assay (Bio-Rad, Hercules, CA).

IL, interleukin; IL-1ra, interleukin-I receptor agonist; PDGF-bb, platelet-derived growth factor-bb; basic FGF, basic fibroblast growth factor; G-CSF, granulocyte colony-stimulating factor; GM-CSF, granulocyte-macrophage colony-stimulating factor; IFN-γ, interferon-gamma; IP-10, interferon-gamma-induced protein; MCP-1, monocyte chemoattractant protein-1; MIP, macrophage inflammatory protein; RANTES, regulated upon activation, normal T-cell expressed and secreted; TNF-α, tumor necrosis factor-alpha; VEGF, vascular endothelial growth factor.

Adapted from Yamato et al. *Blood Adv*. 2024.

| **Supplemental Table 5.** **Antibodies used for flow cytometric diagnosis of TAM** | | | |
| --- | --- | --- | --- |
| Antigen | Clone | Fluorochrome | Source |
| CD45 | 2D1 | PerCP | BD Bioscience |
| CD34 | 581 | PE-Cy7 | Beckmann-Coulter |
| CD117 | 104D2D1 | PE-Cy7 | Beckmann-Coulter |
| HLA-DR | Immu-357 | APC | Beckmann-Coulter |
| CD4 | 13B8.2 | FITC | Beckmann-Coulter |
| CD7 | 8H8.1 | PE | Beckmann-Coulter |
| CD56 | N901 | APC | Beckmann-Coulter |
| CD13 | SJ1D1 | PE | Beckmann-Coulter |
| CD33 | D3HL60.251 | APC | Beckmann-Coulter |
| CD36 | FA6.152 | FITC | Beckmann-Coulter |
| CD41 | P2 | FITC | Beckmann-Coulter |
| CD42b | SZ2 | PE | Beckmann-Coulter |
| CD61 | SZ21 | PE-Cy7 | Beckmann-Coulter |

Adapted from Yamato et al. *Leukemia*. 2021.


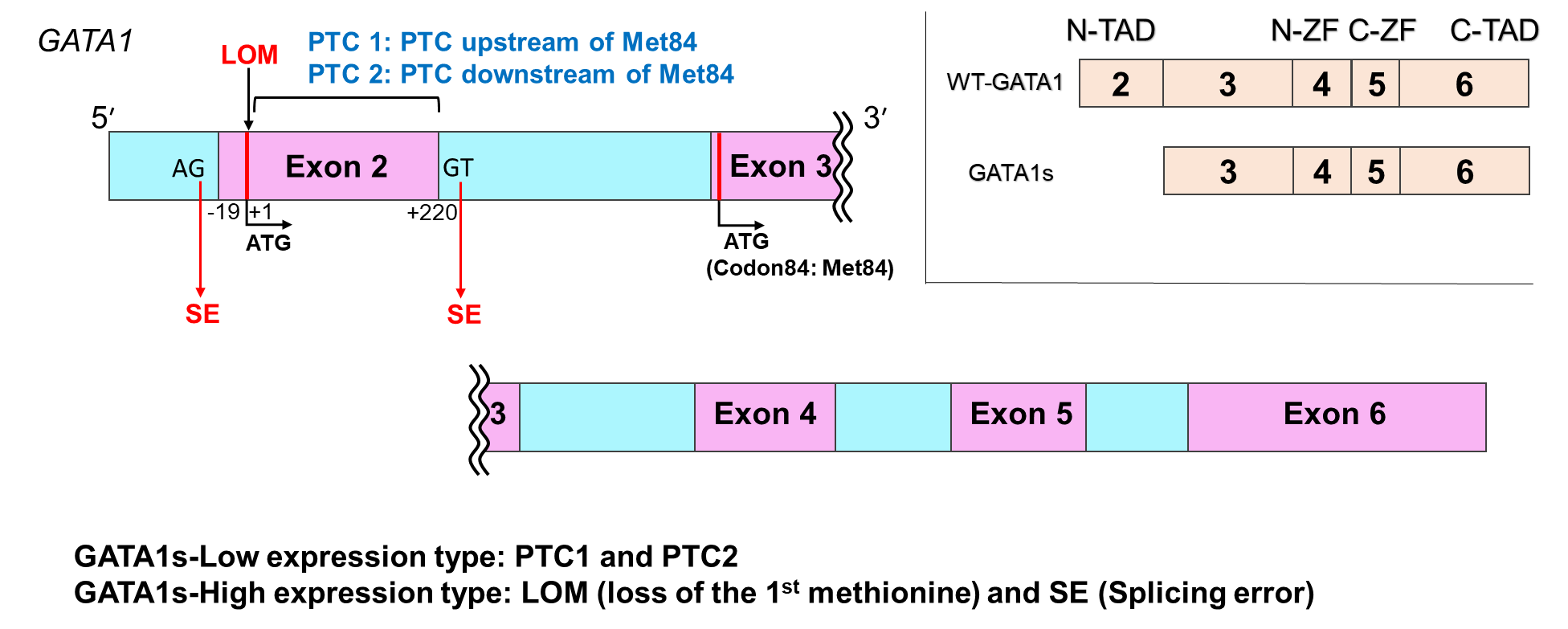


**Supplemental Figure 1. Classification of *GATA1* mutations by predicted expression type**

Mutant *GATA1* alleles alter the expression levels of the translation products. Based on Kanezaki et al. (2010)¹, transcripts encoding the short-form GATA1 protein (GATA1s) were classified into three categories: loss of the first methionine (LOM), splicing error (SE), and premature termination codon (PTC). The PTC group was further subdivided according to the position of the introduced stop codon: PTC type 1, with a PTC occurring before the second methionine at codon 84, and PTC type 2, with a PTC occurring after codon 84. Thus, PTC1 and PTC2 generate premature termination codons before and after the second translation start codon (Met84), respectively. Low-expression–type mutations were defined as PTC (both PTC1 and PTC2), whereas high-expression–type mutations were defined as LOM or SE. Although some PTC mutations located at the 3′ region of exon 2 (previously termed PTC type 1–3′) may induce efficient skipping of exon 2 and lead to relatively high GATA1s expression, others do not,¹ making functional prediction inconsistent. To ensure clarity and analytical consistency, all PTC mutations were grouped into a single “PTC” category and classified as GATA1s-low.

Modified from Yamato et al., *Leukemia*, 2021².

References:

1. Kanezaki R, Toki T, Terui K, et al. Down syndrome and GATA1 mutations in transient abnormal myeloproliferative disorder: mutation classes correlate with progression to myeloid leukemia. *Blood*. 2010.
2. Yamato G, Deguchi T, Terui K, et al. Predictive factors for the development of leukemia in patients with transient abnormal myelopoiesis and Down syndrome. *Leukemia*. 2021;35(5):1480-1484.

**
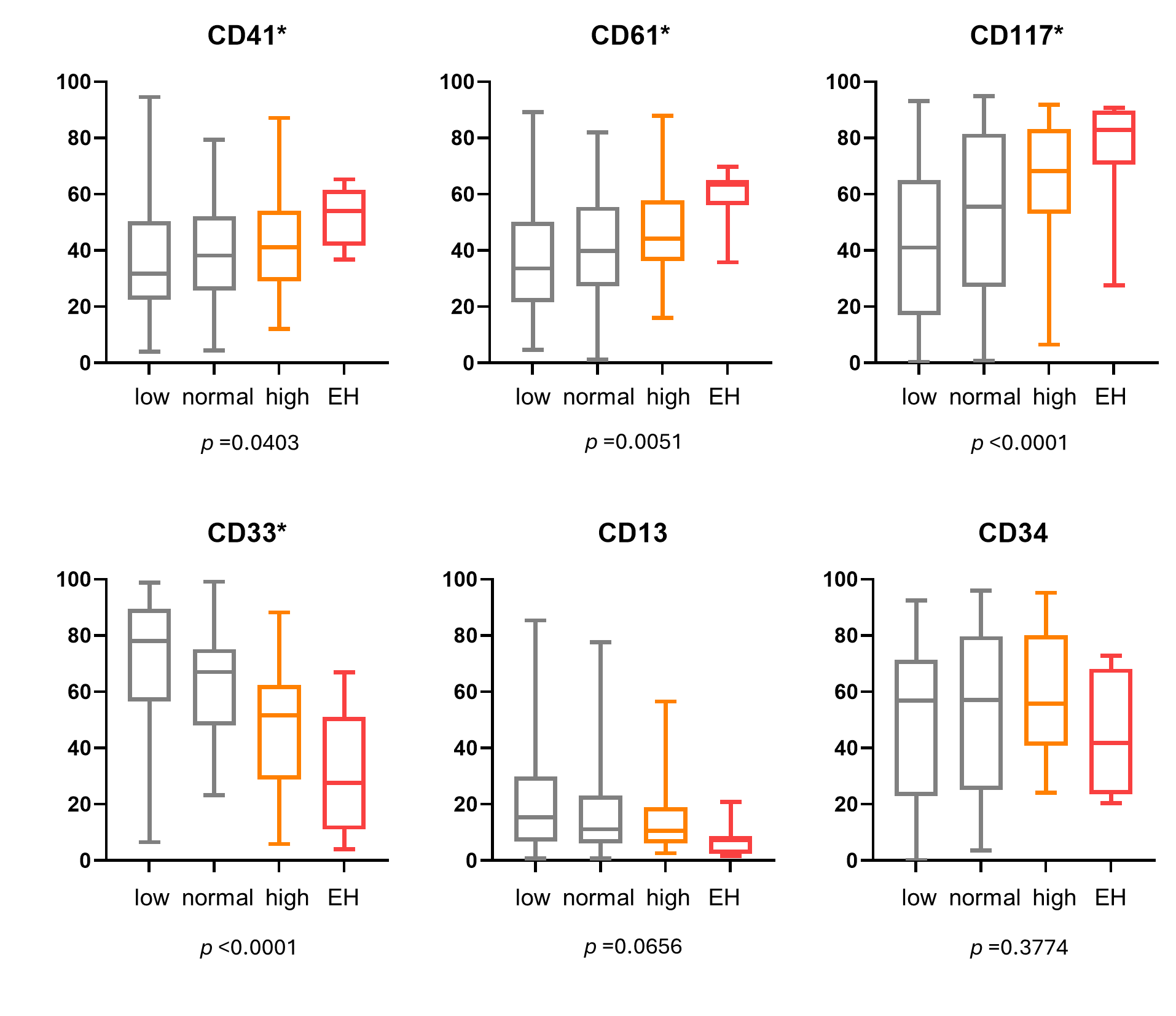
**

**Supplemental Figure 2. Surface marker expression profiles in blast cells**

Bar plots showing the proportion of blast cells expressing each surface antigen across platelet count groups (extremely high (EH), high, normal, and low). Asterisks indicate statistically significant differences.

**
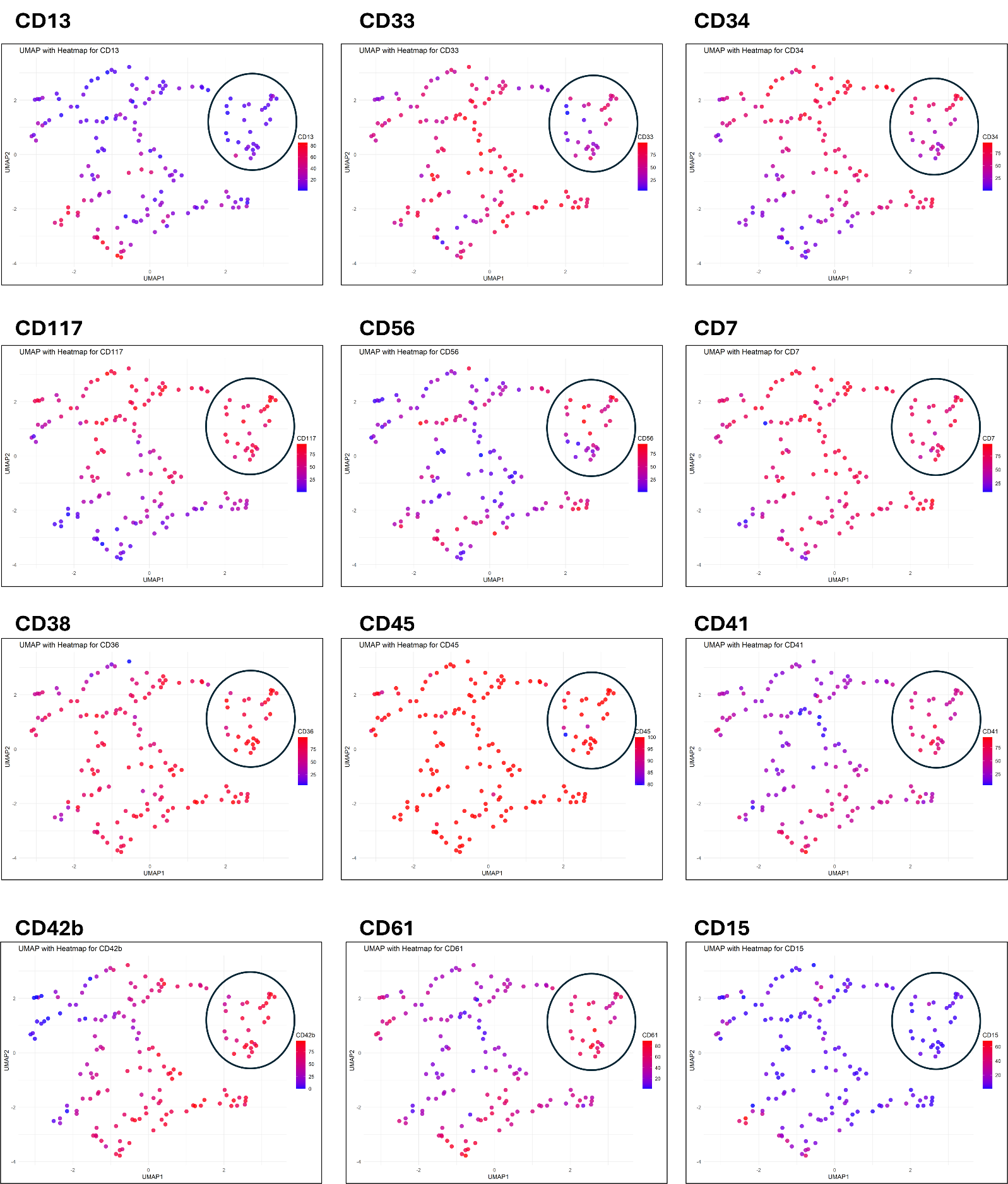
**

**Supplemental Figure 3. Surface marker expression profiles on blast cells**

UMAP plot of 164 TAM patients based on the expression of six antigens (CD7, CD117, CD13, CD33, CD41, and CD61). Expression levels of each antigen are overlaid on UMAP coordinates, with red indicating high and blue indicating low expression. The circle highlights a loosely defined cluster of patients with extremely high (EH) platelet counts, suggesting partial similarity in their immunophenotypic profiles.

**
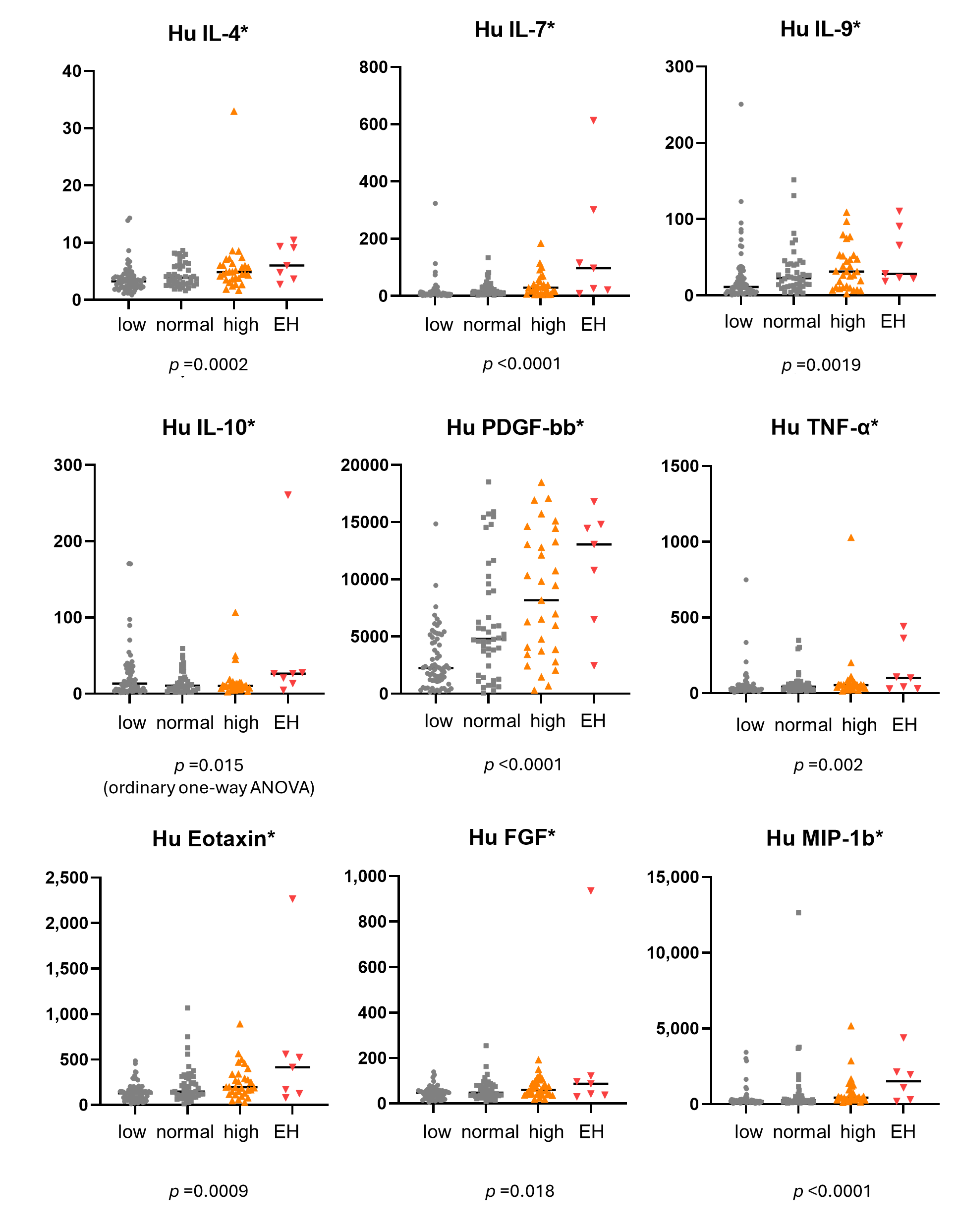
**

**
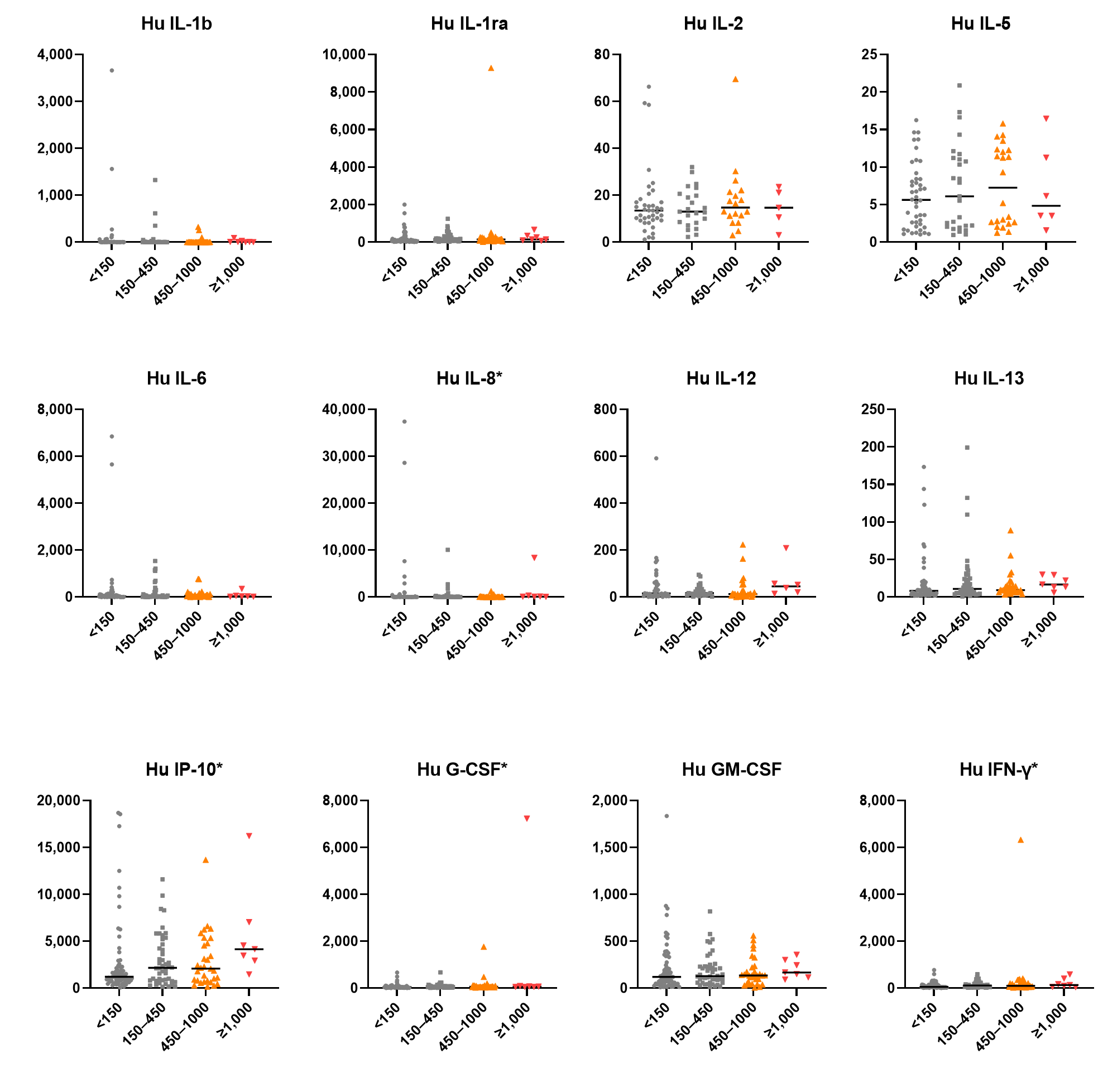
**

**Supplemental Figure 4. Association between platelet counts and cytokine levels in TAM patients**

Bar plots showing serum cytokine levels for individual patients stratified into four groups based on platelet counts at diagnosis: extremely high (EH; ≥1,000×10⁹/L), high (450–1,000×10⁹/L), normal (150–450×10⁹/L), and low (<150×10⁹/L). Statistical comparisons were performed using the Kruskal–Wallis test or one-way ANOVA, as appropriate. Asterisks denote statistically significant differences.


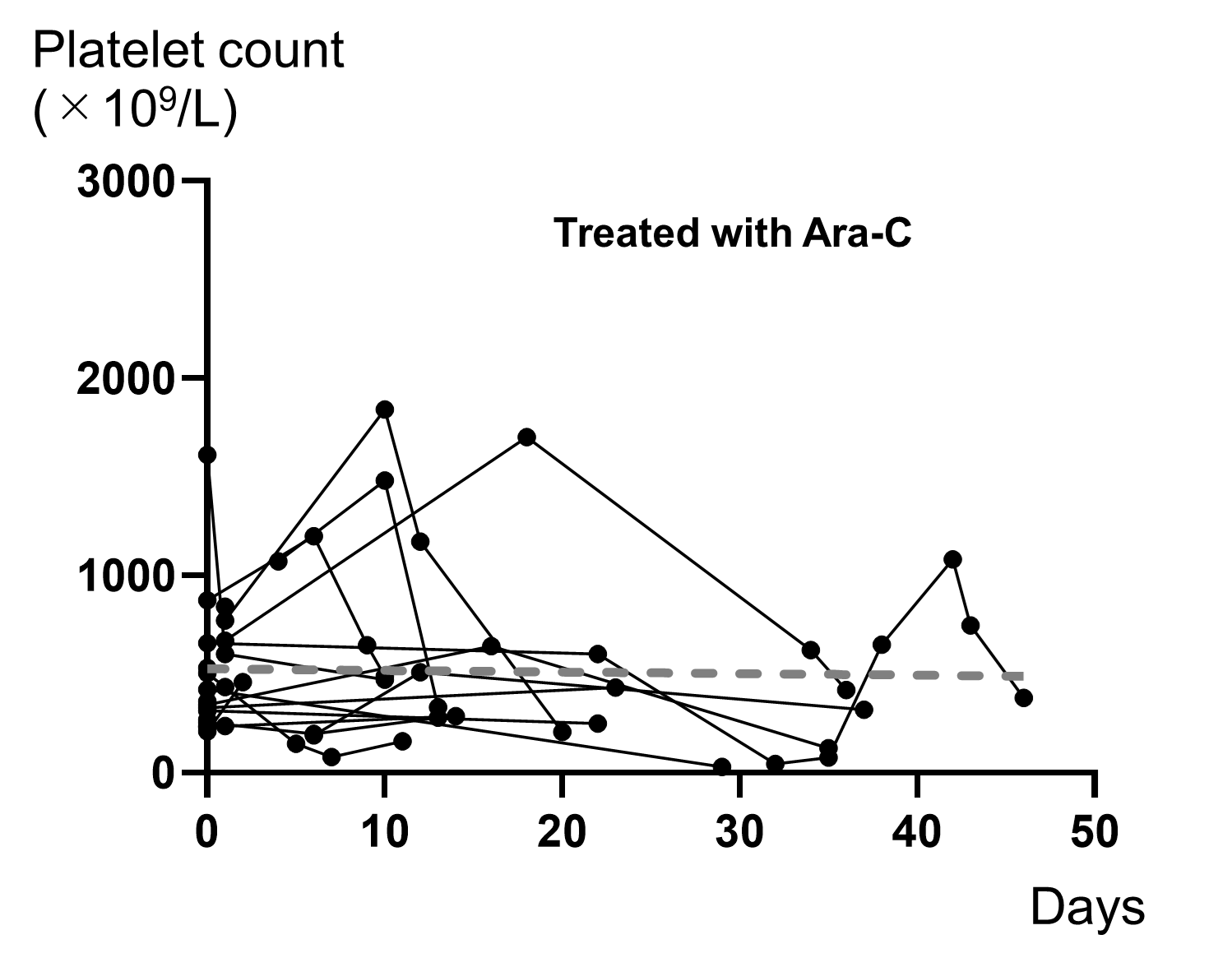


**Supplemental Figure 5. Sequential changes in platelet counts in TAM patients treated with Ara-C**

Among patients with TAM for whom longitudinal platelet counts were available, 21 patients were treated with cytarabine (Ara-C). Solid black lines represent longitudinal changes in platelet counts for individual patients, and gray dashed lines indicate simple linear regression lines.


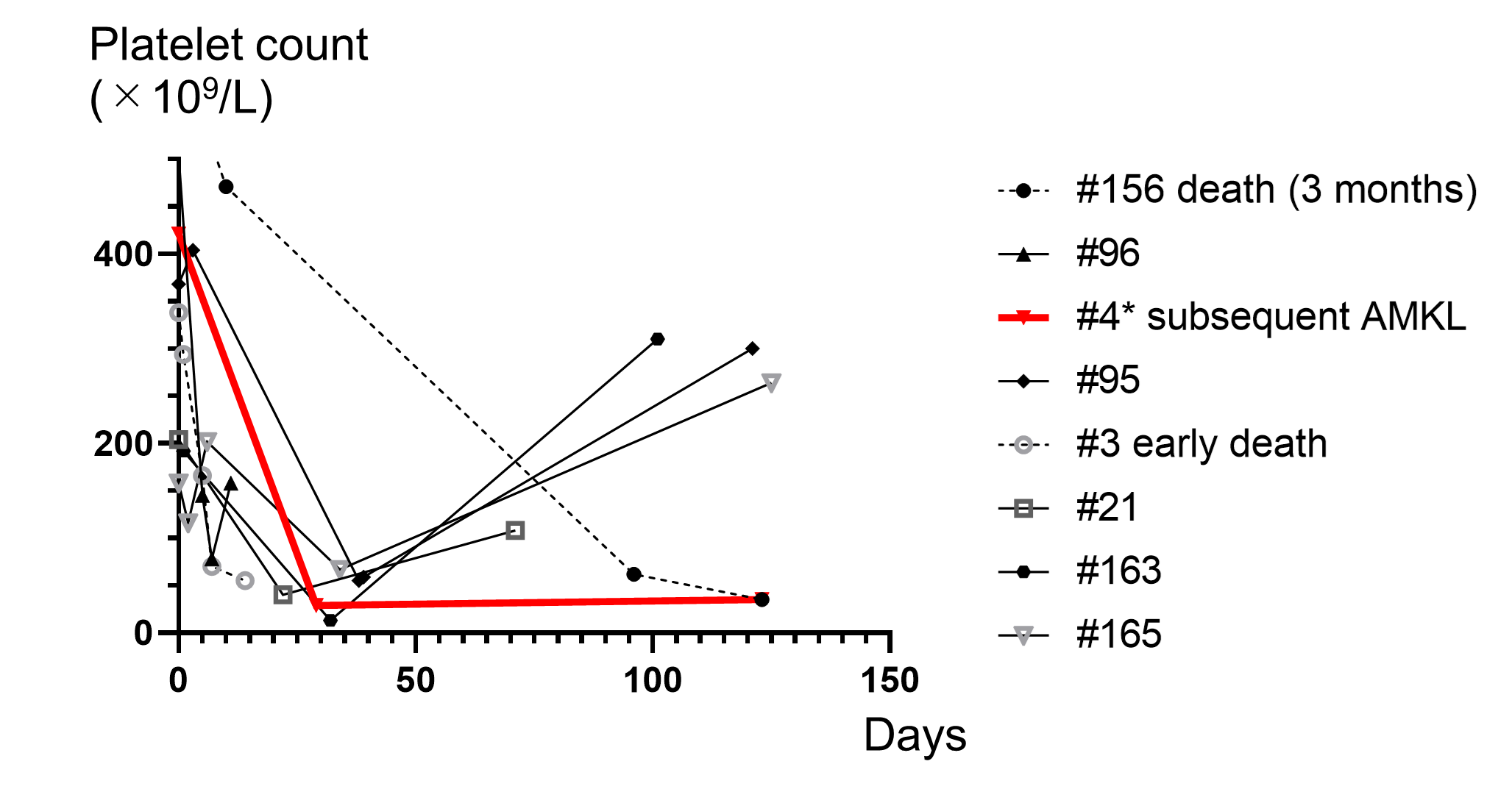


**Supplemental Figure 6. Sequential changes in platelet count in TAM patients who developed delayed thrombocytopenia**

Eight patients initially presented with normal or high platelet counts at diagnosis but later developed delayed-onset thrombocytopenia. Platelet levels dropped significantly during the second to third week and gradually recovered within approximately three months. Most cases recovered spontaneously, whereas persistent thrombocytopenia was associated with leukemic transformation (case #4: red line) or fatal outcomes (cases #3 and #156: dashed line).

**References in the Supplemental Methods**

1. Yamato G, Deguchi T, Terui K, Toki T, Watanabe T, Imaizumi T*, et al.* Predictive factors for the development of leukemia in patients with transient abnormal myelopoiesis and Down syndrome. *Leukemia* 2021 May; **35**(5)**:** 1480-1484.

2. Terui K, Toki T, Taga T, Iwamoto S, Miyamura T, Hasegawa D*, et al.* Highly sensitive detection of GATA1 mutations in patients with myeloid leukemia associated with Down syndrome by combining Sanger and targeted next generation sequencing. *Genes Chromosomes Cancer* 2020 Mar; **59**(3)**:** 160-167.

3. Kanezaki R, Toki T, Terui K, Xu G, Wang R, Shimada A*, et al.* Down syndrome and GATA1 mutations in transient abnormal myeloproliferative disorder: mutation classes correlate with progression to myeloid leukemia. *Blood* 2010 Aug 20.

4. Yamato G, Tsumura Y, Muramatsu H, Shimada A, Imaizumi T, Tsukagoshi H*, et al.* Cytokine profiling in 128 patients with transient abnormal myelopoiesis: a report from the JPLSG TAM-10 trial. *Blood Advances* 2024; **8**(12)**:** 3120-3129.

5. Shimizu R, Takahashi S, Ohneda K, Engel JD, Yamamoto M. In vivo requirements for GATA-1 functional domains during primitive and definitive erythropoiesis. *Embo j* 2001 Sep 17; **20**(18)**:** 5250-5260.

6. Takahashi S, Onodera K, Motohashi H, Suwabe N, Hayashi N, Yanai N*, et al.* Arrest in primitive erythroid cell development caused by promoter-specific disruption of the GATA-1 gene. *J Biol Chem* 1997 May 9; **272**(19)**:** 12611-12615.

7. Ishihara D, Hasegawa A, Hirano I, Engel JD, Yamamoto M, Shimizu R. The abundance of the short GATA1 isoform affects megakaryocyte differentiation and leukemic predisposition in mice. *Exp Hematol Oncol* 2024 Feb 26; **13**(1)**:** 24.
